# Health Literacy and Type 1 Diabetes Mellitus: A Mixed Method Study

**DOI:** 10.3928/24748307-20260311-01

**Published:** 2026-07

**Authors:** Anne-Sofie Kortegaard, Anette Andersen, Anne Emilie Aastrup, Helle Rokkedal Sandersen, Annesofie Lunde Jensen

**Affiliations:** a Steno Diabetes Center Aarhus, Aarhus University Hospital, Aarhus N; b Department of Public health, Aarhus University, Aarhus C; c Aarhus University Hospital, A1001 Department of Clinical Medicine, Aarhus N, Denmark.

## Abstract

**Background:**

The increasing prevalence of type 1 diabetes underscores the need for health literacy responsiveness, as effective self-management depends on understanding and using health information. The Ophelia process supports identifying diverse strengths and needs among people with type 1 diabetes.

**Objectives:**

To systematically identify health literacy strengths, needs, and preferences among individuals with type 1 diabetes attending specialized outpatient care using a mixed methods approach guided by phase 1, step 1 and 2 of the Ophelia process.

**Methods:**

Adults (≥18 years) with type 1 diabetes or subtypes (MODY/LADA) at a Danish specialist diabetes center were invited to complete a survey including the Health Literacy Questionnaire (HLQ) and three scales from the electronic HLQ (eHLQ). Cluster analysis and in-depth interview were integrated for the development of representative vignettes.

**Key Results:**

Of 1,193 eligible patients, 238 participated (response rate 19.9%). The mean age was 54.2 years and 52% were men. HLQ and eHLQ scores revealed a wide diversity in health literacy strengths and needs. Health literacy profiles ranged from higher health literacy with active self-management to lower health literacy with low engagement and high reliance on family support. Vignettes vividly illustrated archetypal patient experiences, supporting a tailored understanding of health literacy challenges in clinical practice.

**Conclusions:**

Seven distinct health literacy profiles were identified among adults with type 1 diabetes, showing significant variation in health literacy and low health literacy among nearly one fifth, with difficulties navigating the health care system being common. The findings underscore the necessity of developing tailored, person-centered, and health literacy responsive interventions to strengthen diabetes self-management and advance equity in health care access.

## Abstract (plain language summary)

**Plain Language Summary:**This study examined health literacy among adults with type 1 diabetes in Danish outpatient care. Seven health literacy profiles with varying strengths and needs were identified. Nearly one in five experienced low health literacy, particularly in navigating health care. These findings advance insight by highlighting the need for tailored, health literacy responsive care to support equitable diabetes self-management.

In 2025, around 9.5 million people are estimated to live with type 1 diabetes (T1DM) including subtypes (e.g., LADA and MODY), projected to reach 14.7 million prevalent cases by 2050 (Ogle et al., 2025). T1DM results in absent or severely reduced insulin production, risk of diabetes-related complications, and lifelong insulin therapy to maintain glycemic control. Self-management is complex and demanding, often leading to challenges with treatment adherence (Carstensen et al., 2020). As the number of people living with T1DM continues to rise globally, outpatient clinics must support increasingly complex, long-term self-management with limited resources. In this regard, health literacy (HL) is a suitable multidimensional concept, which can be used to inform how consultations, communication, and interventions are organized (Milani et al., 2025). HL is defined by The World Health Organization (WHO) as ‘people's knowledge, confidence, and comfort to access, understand, appraise, remember, and use information about health and health care’ (WHO, 2022). HL is essential for interpreting blood glucose, recalling instructions, and using diabetes technologies (Wagner & Olesen, 2023; Weiss et al., 2020). Few studies report prevalence in T1DM or subtypes e.g., LADA, with 10% to 17% inadequate HL (Gomes et al., 2020; Kasper et al., 2017; (Olesen et al., 2017). To address diverse HL needs, health care systems should deliver tailored education and support to enable equitable engagement and improved outcomes in a cost-effective manner (Ahola & Groop, 2013; ElSayed et al., 2025). This enhances the system's HL responsiveness, defined as the ability to deliver equitable access and engagement tailored to HL needs (WHO, 2022).

Epidemiological studies dominated HL research, reporting averages, and sociodemographic (Abdullah et al., 2019). Strengths-based and solution-oriented approaches such as the Optimizing Health Literacy and Access (Ophelia) process, and recommended by WHO, aim to identify HL diversities to tailor actions via cluster analysis, qualitative methods, codesign, and vignettes (Cheng et al., 2024; Osborne et al., 2022). The aim of this study was to systematically identify HL strengths, needs, and preferences among individuals with T1DM according to the Ophelia process phase 1, step 1 and 2.

## Methods

### Study Design and Setting

**Figure 1.**
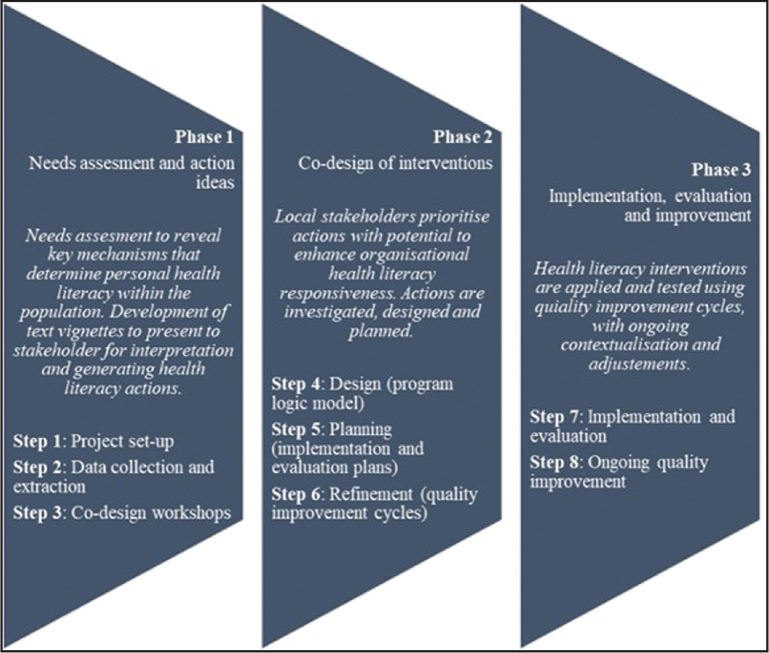
Descriptions of the three phases and eight steps of the Ophelia (Optimizing Health Literacy and Access) process.

This mixed method study was conducted at a Danish outpatient clinic, treating approximately 3,000 individuals with T1DM or related subgroups (Anonymous, 2023). Data were collected and analyzed sequentially in an explanatory mixed methods design (Creswell & Plano Clark, 2022). The study is embedded in a larger project guided by the Ophelia process, with three phases supported by eight principles presented in **Figure 1**. The present study addressed phase 1, steps 1 and 2, conducting a needs assessment to develop text vignettes (see **Table A**) (Osborne et al., 2022). Reporting adheres to GROVE (Guideline for RepOrting Vignette Experiments), as this study develops data-driven vignettes representing archetypal HL profiles for clinical application, following an experimental vignette development process (Hillen et al., 2025).

**Table A.** T1DM and Subtypes Cluster Analysis Interpretation

Based on the scores for each cluster, the health literacy patterns across the seven clusters can be interpreted as summarized in the table below. HLQ scales are presented first, and then eHLQ scales at the end in each “Interpretation” column.		
Across all clusters, participants were most often born in Denmark (between 90.9–100.0%), and not living alone (between 22.2–31.9%). The majority had lived with diabetes for more than five years (between 75.6–100.0%), used technology to support their diabetes management (72.7–100.0%), and had at least one additional health condition beyond diabetes (1.0–2.4).		

**Cluster**	**Interpretation**	**Sociodemographic and clinical characteristics**

A	Individuals in this cluster are proactive and capable of taking responsibility for their own health. They actively seek out the information and support they need (scales 3 and 6). They are able to advocate for their health needs and navigate the healthcare system to locate relevant information and services. They also maintain good relationships with one or more healthcare providers with whom they can collaborate effectively (scales 1 and 7). They are skilled at finding reliable and relevant health information and understanding it well enough to know how to act upon it (scales 2, 8, and 9). Furthermore, they have a social network that provides adequate support when needed (scale 4).	Demographic data indicate that a typical individual in this cluster is a middle-aged (51 ± 21 years) man or woman (56% men). They are often well educated (50% have three years or more of postsecondary education) and are either retired or working full-time (33% and 31%, respectively). Individuals in this cluster are slightly overweight (BMI 26 ± 5 kg/m^2^), engage in light or moderate-to-vigorous physical activity (42% and 47%, respectively), and typically always follow a diabetes-friendly diet (69%). They rarely or never feel lonely (78%) and often have the social support they need (81%). Most are well regulated regarding glycemic control (HbA1c 52 ± 9 mmol/mol), and have at least one additional health condition besides diabetes (1.2 ± 0.6).
	The main challenges experienced by individuals in this cluster relate to staying up to date with new knowledge, comparing information from multiple sources, deciding what is best for themselves, and critically appraising health information (scale 5).	
	Individuals in this cluster perceive technology as useful for managing their health (eHLQ scale 5). They are able to use technology to read, write, and remember information and understand the context-specific language and systems used in various digital platforms, which they also experience as being adaptable to their individual needs (eHLQ scales 1 and 7).

**Table:** 

B	Individuals in this cluster perceive that they have sufficient knowledge to make decisions about their own health and understand written health information and instructions from healthcare professionals well enough to follow them (scales 2 and 9). They have a trusting relationship with at least one healthcare professional and feel that they have control in interactions with healthcare providers, asking clarifying questions when in doubt (scales 1 and 6). They are able to advocate for themselves within the healthcare system, locate the services they need, and receive support from their social network when necessary (scales 4 and 7). They actively seek information from various sources but may experience challenges distinguishing between high- and low-quality information. For example, they may not always ask healthcare professionals to assess the quality of the information they find or compare information obtained from different sources (scales 5 and 8). Setting personal health goals and planning health-promoting activities can also be challenging for these individuals, as other aspects of life may take precedence over health priorities (scale 3).	Demographic data indicate that a typical individual in this cluster is a middle-aged (56 ± 19 years) man or woman (54% men). They are generally well educated (54% have three years or more of postsecondary education) and are either retired or working full-time (39% and 27%, respectively). Individuals in this cluster are slightly overweight (BMI 26 ± 6 kg/m^2^), engage in light or moderate-to-vigorous physical activity (44% and 49%, respectively), and typically always follow a diabetes-friendly diet (78%). They rarely or never feel lonely (66%) and often receive the social support they need (78%). Most have an HbA1c of 57 ± 15 mmol/mol, and have at least one additional health condition besides diabetes (1.5 ± 0.9).
	These individuals generally find technology meaningful for managing their health, but they may face some limitations in understanding or using it effectively (eHLQ scales 1 and 5). They also experience that available digital health services are not always adaptable to individual needs - for example, when dealing with impaired vision (eHLQ scales 1, 5, and 7).	

C	Individuals in this cluster are able to seek out and understand health information well enough to know how to act on it and typically have a social network that can provide support when needed (scales 4, 8, and 9). They are capable of locating relevant healthcare services and advocating for themselves when interacting with healthcare professionals, with whom they engage actively and experience a high degree of control (scales 6 and 7).	Demographic data indicate that a typical individual in this cluster is a middle-aged (53 ± 17 years) man or woman (52% men). They typically have a vocational or medium-length postsecondary education (22% and 39%, respectively) and are either retired or working full-time (37% and 30%, respectively). Individuals in this cluster are slightly overweight (BMI 27 ± 5 kg/m^2^), engage in light or moderate-to-vigorous physical activity (46% and 48%, respectively), and typically always follow a diabetes-friendly diet (70%). They rarely or never feel lonely (63%) and often have the social support they need (74%). Most have an HbA1c
	They do not necessarily have a trusted relationship with a known healthcare professional, which may challenge their ability to make health-related decisions (scale 1). They do not always feel

**Table:** 

	that they have sufficient information to make informed decisions about their own health and may find it difficult to take responsibility for and prioritize their health management (scales 2 and 3). They may also experience difficulties distinguishing between high- and low-quality health information and may rely on others for assistance in evaluating it (scale 5).	of 59 ± 10 mmol/mol, and have at least one additional health condition besides diabetes (1.4 ± 0.8).
	These individuals generally understand and use technology effectively and find it meaningful for monitoring their health and communicating with healthcare professionals (eHLQ scales 1 and 5). However, they may experience that available digital health services are not always adaptable to individual needs - for instance, in cases of visual impairment (eHLQ scale 7).	

D	This cluster comprises 30.3% of the total sample. Individuals in this cluster generally perceive that they have sufficient knowledge to manage their own health and tend to engage actively in maintaining it, viewing health management as their personal responsibility (scales 2 and 3). They understand written health information and instructions from healthcare professionals well enough to act on them (scale 9). They are typically able to advocate for themselves within the healthcare system and have a known healthcare professional with whom they can communicate openly and ask clarifying questions (scales 1 and 6).	Demographic data indicate that a typical individual in this cluster is a middle-aged (51 ± 18 years) man or woman (50% men).
	These individuals do not always have access to social support and may find it difficult to locate reliable health information, understand available services, or know what they are entitled to within the healthcare system (scales 4, 7, and 8). Some may also find it challenging to assess whether health information is of good or poor quality unless they receive help from others (scale 5).	They commonly have a short-cycle higher education (33%) and are either retired or working full-time (37% and 30%, respectively). Individuals in this cluster are likely to be slightly overweight (BMI 25 ± 5 kg/m^2^), engage in light or moderate-to-vigorous physical activity (39% and 53%, respectively), and typically always follow a diabetes-friendly diet (70%). They rarely or never feel lonely (63%) and often receive the social support they need (60%). Most have an HbA1c of 53 ± 10 mmol/mol, and have at least one additional health condition besides diabetes (1.6 ± 1.9).
	Individuals in this cluster consider the use of technology and digital services to be useful in relation to their health and treatment (eHLQ scale 5). However, they often find it difficult to use technology and to understand the structure and context-specific language of different digital systems, which they

**Table:** 

	also experience as not being well adapted to individual needs - for example, in cases of impaired vision (eHLQ scales 1 and 7).	

E	Individuals in this cluster typically receive strong support from their social network and are good at proactively engaging in their own health and planning activities that promote health (scales 3 and 4). They do not actively seek information from multiple sources but do not generally feel limited in their ability to distinguish between good- and poor-quality information (scales 5 and 8). They do not have a trusted relationship with a known healthcare professional who can support them in making decisions about their health (scale 1). In interactions with healthcare professionals, they tend to adopt a more passive approach and, for example, may not ask questions when in doubt or critically consider whether what is being offered fits their own needs (scale 6). They often experience difficulties advocating for themselves, identifying relevant services in the healthcare system, and are frequently unaware of their rights (scale 7). Some feel they have insufficient health information to manage their condition, and written health information may be difficult to understand, which makes it challenging to complete forms, such as questionnaires (scales 2 and 9).	Demographic data indicate that a typical individual in this cluster is an older (56 ± 17 years) man (61% men). They usually have a vocational education (44%) and are either working full-time or receiving early retirement benefits (28% and 33%, respectively).
	Individuals in this cluster consider the use of technology and digital services meaningful in relation to their health and treatment and are generally able to understand and use the digital services made available to them (eHLQ scales 1 and 5). However, they often experience that digital solutions are not adapted to their individual needs—for example, in the case of impaired vision (eHLQ scale 7).	Individuals in this cluster are slightly overweight (BMI 27 ± 5 kg/m^2^), engage in light or moderate-to-vigorous physical activity (44% and 56%, respectively), and typically always follow a diabetes-friendly diet (61%). They rarely or never feel lonely (67%) and often receive the social support they need (56%). Most have an HbA1c of 55 ± 5 mmol/mol, and have at least one additional health condition besides diabetes (1.7 ± 0.9).

F	Individuals in this cluster generally perceive that they have sufficient information to manage their own health and feel that they have control in their interactions with healthcare professionals, where they ask questions when in doubt and advocate for themselves (scales 2 and 6). They have a reasonable understanding of their rights, are typically able to locate relevant healthcare services, and understand health information well	Demographic data indicate that a typical individual in this cluster is an older (64 ± 14 years) woman (41% men). They commonly have a medium-length postsecondary education (41%) and are retired, working full-time, or receiving early retirement benefits (50%, 18%, and 18%, respectively). Individuals in this cluster are overweight (BMI 29 ± 5 kg/m^2^), engage primarily in light physical activity (59%), and always or often follow a

**Table:** 

	enough to follow medication instructions and treatment guidance (scales 7 and 9). However, they often lack a trusted relationship with a known healthcare professional who understands them and can support their decision-making, and several do not have access to social support (scales 1 and 4). They do not typically seek out health information on their own and may find it difficult to understand health information, becoming confused when it is complex or contradictory (scales 5 and 8). They are not always actively engaged in managing their health and may not necessarily perceive it as their personal responsibility (scale 3).	diabetes-friendly diet (50%). They rarely or never feel lonely (55%) and often receive the social support they need (55%).
		Most have an HbA1c of 59 ± 8 mmol/mol. They typically have more than two additional health conditions besides diabetes (2.4 ± 1.2). More than one-fifth of this cluster completed the questionnaire in person (23%), and a few required assistance to respond (9%).
	Individuals in this cluster have limited motivation to use digital services for managing their health and may find it difficult to understand and use available digital solutions in ways that support reading, writing, remembering, or communicating with, for instance, healthcare professionals (eHLQ scales 1 and 5). They also often experience that digital solutions are not adapted to their individual needs—for example, in the case of impaired vision (eHLQ scale 7).	

G	Individuals in this cluster vary in terms of their social support network, the extent to which they have sufficient health information to manage their health, and whether they have a trusting relationship with at least one healthcare professional. They may therefore possess either significant resources or substantial limitations in these areas (scales 1, 2, and 4). Overall, they tend to adopt a passive approach in interactions with healthcare professionals—typically accepting information without asking questions, seldom sharing concerns, not seeking clarification when something is unclear, and generally feeling little sense of control or involvement in decision-making (scale 6). However, some individuals within this cluster do have a positive and trusting relationship with at least one healthcare professional. They are usually not actively engaged in their own health and do not perceive health management as their responsibility, viewing healthcare as something that is done to them rather than with them (scale 3). They have limited	Demographic data indicate that a typical individual in this cluster is an older (74 ± 17 years) man (67% men). Their highest completed education is typically primary school (67%), and they are either retired, working full-time, or receiving early retirement benefits (33%, 33%, and 33%, respectively). Their body weight varies (BMI 24 ± 12 kg/m^2^); they often have a sedentary activity level (67%) and rarely or never follow a diabetes-friendly diet (67%). They rarely or never feel lonely (67%), but some report seldom or never having social support (33%), while others report often receiving support (33%). Individuals in this cluster are generally poorly regulated with respect to glycemic control (HbA1c 73 ± 1 mmol/mol) and typically have at least one additional health condition besides diabetes (1.0 ± 1.7). Most participants in this cluster completed the questionnaire in person (67%), and one-third required assistance to complete it (33%).

**Table:** 

	awareness of available services and their rights within the healthcare system and often find it difficult to advocate for themselves or to get help navigating the system, even though they may have some degree of social network support (scales 7 and 4). Their collaboration with and trust in healthcare professionals as sources of information vary across individuals (scale 1).	
	Regardless of effort, most health information is difficult for them to understand, and encountering contradictory information causes confusion. As a result, they generally do not seek recommended health information and rely on others to offer it to them (scales 5 and 8). Difficulties in understanding written health information also make it hard for them to complete forms or follow treatment and medication instructions (scale 9). Variation exists in the extent to which individuals in this cluster believe they have the information needed to manage their health and make informed decisions (scale 2).	
	They do not use technology to find health information, track their health status, or manage their treatment, and they do not feel comfortable using digital services in communication with healthcare professionals, such as virtual consultations (eHLQ scales 1 and 5). They also perceive that existing technologies cannot be adapted to their abilities or individual needs (eHLQ scale 7).	

### Participants

Data were collected between September 2024 to March 2025. No accepted sample size standards exist, but stable cluster solutions appear with 50 to 100 participants (Osborne et al., 2022). Inclusion criteria were individuals ≥18 years of age attending the outpatient in September 2024 or October 2024, diagnosed with T1DM or subgroups such as MODY or LADA (American Diabetes Association [ADA], 2014). As subgroups are managed within the same outpatient care as individuals with T1DM, pooling them was clinically relevant despite etiological differences in the diagnosis. Participants with cognitive impairment, severe mental illness, or acute critical illness preventing informed consent or survey completion were excluded.

### Measurements

The HLQ was developed and validated for use in a Danish context (Maindal et al., 2016). The HLQ consists of 44 items covering nine distinct scales of HL. Scale 1 to 5 uses response options of *strongly disagree* to *strongly agree* (score range 1 to 4), while scale 6 to 9 uses the response options *cannot do or always difficult* to *always easy* (score range of 1 to 5). eHLQ was validated in a Danish context to measure interaction with digital health services (Kayser et al., 2018). The eHLQ contains 35 items covering seven scales with response options from *strongly disagree* to *strongly agree* (score range of 1 to 4). In this study, scales 1, 5, and 7 were selected (14 items in total), as they specifically assess motivation for, engagement with, and perceived fit of digital health services, therefore they were relevant to clinical insights into technology use. Self-reported sociodemographic data and health data were collected, including assistance with completing the questionnaire. Hemoglobin A1c (HbA1c) values for each participant were collected from the electronic medical record, with a cut-off at 7% (53 mmol/mol), as this is the goal for most adults with diabetes (ADA, 2025).

### Ethical Considerations

In accordance with Danish law, approval by the Danish National Committee on Health Research Ethics is not required for studies based on questionnaire data. All participants provided written consent and were informed of their right to withdraw from the study at any time. Data collection and analysis followed the World Medical Association's Code of Ethics (World Medial Association, 2022), and data were pseudonymized in accordance with the General Data Protection Regulation (Guidance on consent, 2021). The project was registered with the Central Denmark Region (J.no 1-45-70-79-24).

### Quantitative Data

***Quantitative data collection.*** Maximum variation sampling invited all individuals attending the outpatient clinic in September 2024. Participants were approached face-to-face to include hard-to-reach groups. Questionnaires (77 items) were available in Danish, English, Arabic, Somali, and large print and could be completed independently or with assistance. In December 2024, individuals who had not previously been invited received a digital questionnaire (in Danish), with the option to complete it independently or with assistance from a relative, friend, or a member of the research team. This strategy was implemented to increase the sample size while considering resource constraints.

***Statistical analysis.*** Cluster analysis was utilized to capture the diversity within populations by grouping individuals with similar patterns in HLQ and eHLQ (Osborne et al., 2022; Windgassen et al., 2018). Discrete variables were presented as proportions, and continuous variables were presented as means, SD, and 95% confidence interval. As no established cut-off scores for determining inadequate HL measure exist, we applied cut-off scores of ≤2.5 for scale 1 to 5 in the HLQ and scale 1, 5, and 7 in the eHLQ, and ≤3.5 for scale 6 to 9 in the HLQ, based on recommendations from the user manual (Osborne et al., 2022).

**Figure 2.**
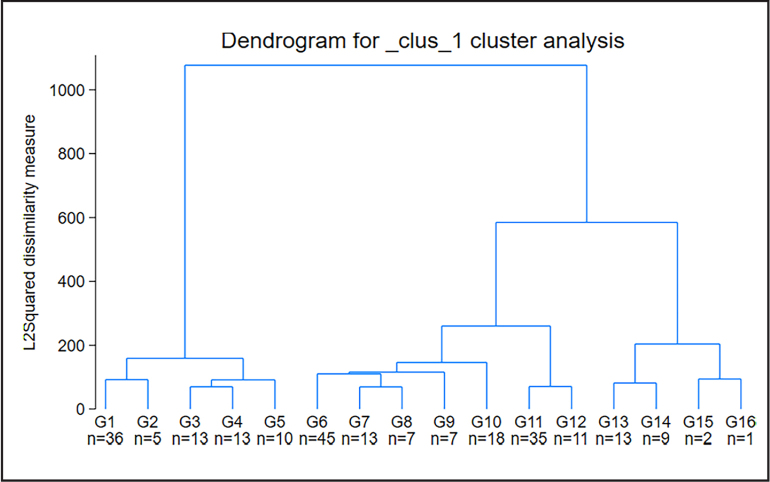
Dendrogram illustrating hierarchical cluster analysis of health literacy profiles based on Health Literacy Questionnaire (HLQ) and electronic HLQ scale scores.

All scores were converted to z-scores to allow comparison of different response ranges, and hierarchical cluster analysis using Ward's linkage was applied (Osborne et al., 2022). Cluster solutions from 3 to 16 clusters were assessed, selecting the optimal number based on dendrogram inspection (**Figure 2**), within-cluster variation (*SD* > 0.6), changes from the parent cluster (>0.5), cluster size, and diversity in sociodemographic and clinical variables. The examination continued until differences in further splits no longer provided new or meaningful patterns in data. Data were summarized in cluster profiles that included HL scores along with sociodemographic data. To visually distinguish levels of scores, a ‘traffic light’ color scheme was used to indicate higher (green), medium (yellow), and lower (red) scores. The identified clusters informed the development of vignettes that represent typical HL profiles observed within the population.

### Qualitative Data

***Qualitative data collection.*** We used Interpretive Description (ID) methodology to ensure practice-oriented and clinically relevant findings (Thorne, 2025). While patient-reported outcomes (PROs) provide important insights into person-centered care (Porth et al., 2024), both clinicians and patients find interpreting quantitative data from PRO measures challenging (Snyder et al., 2017). Data-informed text vignettes, developed by combining data from PRO measures and interviews, have previously been applied to HLQ data and shown to be a recognizable method to enhance interpretation of PRO (Aaby et al., 2020; Kelly et al., 2025). At the end of the survey, participants were invited to take part in an interview focused on their questionnaire responses. Recruitment was purposive, based on the cluster solution and characteristics within each cluster. Interviews were conducted until sufficient data had been collected to inform the development of the vignettes. Study participants selected the location and date of their interview: three were held in the outpatient clinic, while ten were conducted by telephone. Interviews were audiorecorded, and a semi-structured interview guide was used to explore experiences related to HL responses. Durations ranged from 28 to 84 minutes. Open-ended questions included:

- ‘Can you describe a time when someone helped you with your health?’

***ID analysis.*** ID analysis is an inductive process comprising four steps undertaken iteratively (Thorne, 2025). The first author listened to transcribed interviews, summarizing them by cluster, and explored patterns in NVivo (Version 15). The last author reviewed two interviews to support analysis. Qualitative data were then integrated with quantitative findings, and vignettes were drafted. Each vignette was reviewed by all authors, and the final outputs included descriptions of participants' sociodemographic characteristics, HL scores, and participants´ experiences and perspectives regarding HL.

## Results

### Sociodemographic Characteristics

A total of 1,193 eligible individuals were invited to participate in the study, and 238 participants answered the questionnaire, corresponding to a response rate of 19.9 % (**Table 1**). One third (33.6%) were older than age 65 years, and 52.1% were men. 82.8% used diabetes technology (continuous glucose monitoring, flash glucose monitoring, or insulin pump), and 42.4% had HbA1c levels on or below 7% (53 mmol/mol). The majority (95.8%) reported at least one additional health condition. Feelings of loneliness (*often* or *sometimes*) were reported by 32.8%, and 10.9% rarely or never had support when facing problems. Furthermore, participants had a mean body mass index of 26.29 kg/m^2^ (*SD* = 5.05) and a mean HbA1c of 7.2% (*SD* = 3.1), corresponding to 55.61 mmol/mol (*SD* = 10.78).

**Table 1:** Sociodemographic Characteristics for Participants Answered the Questionnaire (*N* = 238)

**Sociodemographic Variable**	**% (n)**

Sex	
Female	47.9 (114)
Male	52.1 (124)

Age (y)	
18–35	22.3 (53)
36–55	21.9 (52)
56–65	22.3 (53)
>65	33.6 (80)

Education completed	
Municipal primary or lower secondary school	6.3 (15)
Upper secondary education	11.3 (27)
Vocational education or short-term further education (2–3 years)	32.4 (77)
3–4 years further education	31.9 (76)
Long-term further education (>4 years)	18.1 (43)

Occupation	
Full-time job	32.4 (77)
Part-time job	8.8 (21)
Student	10.5 (25)
Pensioner including missing	31.9 (76)
Unemployed or stay-at-home	2.9 (7)
Early retirement, long-time illness, receive social security	13.5 (32)

Understand Danish	
Yes	97.1 (231)
Little or no	1.3 (3)
Missing data	1.7 (4)

Physical activity (SGPALS)	
Inactive	7.1 (17)
Some light	43.7 (104)
Minimum 2–3 hours/week	47.5 (113)
Missing data	1.7 (4)

Eating a diabetes friendly diet	
Never or rarely	9.7 (23)
Sometimes	21.0 (50)
Always or often	67.7 (161)
Missing data	1.7 (4)

Feeling alone	
Often	9.7 (23)
Sometimes	23.1 (55)
Rarely or no	65.1 (155)
Missing	2.1 (5)

Having support	
No or rarely	10.9 (26)
Sometimes	18.9 (45)
Often	67.7 (161)
Missing	2.5 (6)

**Table:** 

**Sociodemographic Variable**	**% (n)**

Type 1 diabetes	95 (226)

Diabetes duration	
<5 years	18.1 (43)
5–10 years	8.8 (21)
>10 years	71.4 (170)
Missing data	1.7 (4)

Using technology for monitoring/treating diabetes (CGM, FGM, pump)	82.8 (197)

HbA1c	
≤7% (53 mmol/mol)	42.4 (101)
>7% (53 mmol/mol)	49.6 (118)
Missing data	8 (19)

Other conditions (cardiovascular diseases, cancer, mental illness, late complications)	
None	4.2 (10)
One more than diabetes	56.3 (134)
Two or more than diabetes	39.5 (94)

Note. CGM, continuous glucose monitoring; FGM, flash glucose monitoring; HbA1c, hemoglobin A1C; SMBG, Self-Monitoring of Blood Glucose; SGPALS, Saltin-Grimby Physical Activity Level Scale.

### HLQ and eHLQ Mean Scores

**Table 2** shows mean scores and proportions with inadequate scores within each HL scale. Participants reported moderate-high HL. For the HLQ scales, the highest score was observed for ‘Feeling understood and supported by health care providers’ (mean 3.24; standard deviation [*SD*] 0.61) and ‘Ability to actively engage with health care providers’ (mean 3.88; *SD =* 0.58). The lowest score was in ‘Navigating the health care system’ (mean 3.57; *SD* = 0.66), which also had the highest proportion of participants with inadequate health literacy (43.8%). Mean scores on the eHLQ scales ranged from 2.90 to 3.18, with lowest scores for ‘Digital services that suit individual needs’ (mean 2.90; *SD =* 0.68).

**Table 2:** Mean Scores for the Nine Health Literacy Questionnaire Scales and the Three Ehealth Literacy Questionnaire Scales (*N* = 238)

**HLQ Scale^a^**	**Mean (*SD*) [95 % CI]**	**Proportion with Inadequate Score, % (*n*)**

1. Feeling understood and supported by health care providers	3.24 (0.61) [3.16; 3.31]	8.4 (21)
2. Having sufficient information to manage my health	3.10 (0.56) [3.02; 3.17]	10.1 (26)
3. Actively managing my health	2.92 (0.55) [2.85; 2.99]	16.7 (43)
4. Social support for health	3.00 (0.59) [2.92; 3.08]	16.7 (43)
5. Appraisal of health information	2.79 (0.61) [2.71; 2.87]	26.0 (67)
6. Ability to actively engage with health care providers	3.88 (0.78) [3.78; 3.98]	24.4 (63)
7. Navigating the healthcare system	3.57 (0.66) [3.49; 3.66]	43.8 (113)
8. Ability to find good health information	3.77 (0.67) [3.69; 3.86]	27.5 (71)
9. Understanding health information well enough to know what to do	3.88 (0.64) [3.80; 3.96]	18.6 (48)

**eHLQ scale^a^**		

1. Using technology to process health information	3.08 (0.74) [2.99; 3.18]	14.3 (37)
5. Motivated to engage with digital services	3.18 (0.73) [3.09; 3.27]	12.4 (32)
7. Digital services that suit individual needs	2.90 (0.68) [2.82; 2.99]	22.5 (58)

Note. There were no missing data.

a Score range 1 (*lowest*)-4 (*highest*) for HLQ scales 1–5 and eHLQ; 1 (*lowest*)-5 (*highest*) for HLQ scales 6–9. CI = confidence interval; e = electronic; HLQ = Health Literacy Questionnaire.

### Cluster Analysis

The cluster analysis revealed an overall pattern ranging from higher HL (Cluster A) to lower HL (Cluster G) (**Table B**). The number of individuals in each cluster varied from 3 to 46.

**Table B:** Seven health literacy profiles identified through cluster analysis and associated demographic characteristics (N = 238). Data are % (N) unless otherwise specified.

**Cluster name**	A	B	C	D	E	F	G
Participants	15.1 (36)	17.2 (41)	19.3 (46)	30.3 (72)	7.6 (18)	9.2 (22)	1.3 (3)

**HLQ mean (SD) score within cluster**							

1. Feeling understood and supported by healthcare providers	3.86 (0.35)	3.76 (0.43)	3.07 (0.33)	3.08 (0.40)	2.56 (0.51)	2.73 (0.55)	2.67 (1.53)
2. Having sufficient information to manage my health	3.56 (0.50)	3.51 (0.51)	2.89 (0.32)	3.00 (0.38)	2.61 (0.50)	2.77 (0.61)	2.67 (1.53)
3. Actively managing my health	3.42 (0.50)	2.90 (0.30)	2.70 (0.47)	2.99 (0.49)	3.00 (0.34)	2.50 (0.60)	1.33 (0.58)
4. Social support for health	3.58 (0.50)	3.24 (0.54)	3.00 (0.30)	2.68 (0.53)	3.00 (0.34)	2.64 (0.49)	3.00 (1.73)
5. Appraisal of health information	3.19 (0.53)	3.02 (0.35)	2.59 (0.62)	2.85 (0.49)	2.83 (0.38)	2.09 (0.61)	1.33 (0.58)
6. Ability to actively engage with healthcare providers	4.72 (0.45)	4.27 (0.45)	4.04 (0.42)	3.57 (0.65)	2.83 (0.38)	3.64 (0.73)	1.67 (1.15)
7. Navigating the healthcare system	4.19 (0.53)	3.95 (0.44)	4.04 (0.21)	3.13 (0.37)	2.94 (0.24)	3.09 (0.43)	1.67 (0.58)
8. Ability to find good health information	4.25 (0.50)	4.17 (0.50)	4.02 (0.26)	3.71 (0.52)	2.94 (0.24)	2.91 (0.53)	1.67 (0.58)
9. Understanding health information well enough to know what to do	4.31 (0.62)	4.27 (0.45)	4.02 (0.15)	3.79 (0.41)	3.11 (0.32)	3.41 (0.73)	1.33 (0.58)

**eHLQ mean (SD) score within cluster**							

1. Using technology to process health information	3.44 (0.56)	2.95 (0.81)	3.26 (0.53)	3.19 (0.60)	3.22 (0.43)	2.18 (0.80)	1.00 (0.00)
5. Motivated to engage with digital services	3.75 (0.44)	3.07 (0.69)	3.15 (0.63)	3.35 (0.56)	3.22 (0.47)	2.23 (0.61)	1.00 (0.00)
7. Digital services that suit individual needs	3.64 (0.49)	2.93 (0.47)	2.89 (0.43)	2.94 (0.63)	2.72 (0.46)	1.96 (0.38)	1.00 (0.00)

**Sociodemographic characteristics**							

Mean (SD) age (years)	51.47 (20.91)	55.46 (18.75)	53.33 (17.43)	51.32 (17.77)	55.78 (16.54)	63.64 (14.17)	73.67 (17.24)
Men	55.6 (20)	53.7 (22)	52.2 (24)	50.0 (36)	61.1 (11)	40.9 (9)	-
Mean (SD) BMI (kg/m^2^)	26.28 (4.47)	26.20 (5.76)	26.75 (4.71)	25.40 (4.94)	26.88 (5.31)	28.52 (4.46)	24.22 (12.13)
Living alone	22.2 (8)	26.8 (11)	28.3 (13)	31.9 (23)	-	27.3 (6)	-
Born in Denmark	94.4 (34)	92.7 (38)	93.5 (43)	95.8 (69)	100.0 (18)	90.9 (20)	100.0 (3)

**Table:** 

Understand Danish	94.4 (34)	100.0 (41)	93.5 (43)	98.6 (71)	100.0 (18)	95.5 (21)	100.0 (3)

**Highest education completed**							

Municipal primary or lower secondary school, upper secondary school, or vocational education	44.4 (16)	36.6 (15)	37.0 (17)	31.9 (23)	55.6 (10)	13.6 (9)	-
≥ 3 years further education	50.0 (18)	53.7 (22)	54.3 (25)	52.8 (38)	27.8 (5)	50.0 (11)	-

**Occupation**							

Working full-time	30.6 (11)	26.8 (11)	30.4 (14)	43.1 (31)	27.8 (5)	18.2 (4)	-
Pensioner	33.3 (12)	39.0 (16)	37.0 (17)	18.1 (13)	22.2 (4)	50.0 (11)	-

**Health behavior**							

Physically inactive or some light physical activity	47.2 (17)	48.8 (20)	52.2 (24)	45.8 (33)	44.4 (8)	72.7 (16)	100.0 (3)
Always/often eating a diabetes friendly diet	69.4 (25)	78.0 (32)	69.6 (32)	69.4 (50)	61.1 (11)	50.0 (11)	-

**Loneliness**							

Often or sometimes unwanted alone	19.4 (7)	31.7 (13)	34.8 (16)	36.1 (26)	33.3 (6)	45.5 (10)	-
Never/rarely/sometimes having social support	16.7 (6)	19.5 (8)	23.9 (11)	38.9 (28)	38.9 (7)	45.5 (10)	-

**Medical conditions**							

							
Mean (SD) HbA1c, %; SD (mmol/mol; SD)	6.9;3.0 (52.00;8.85)	7.4;3.5 (57.21;14.89)	7.6;3.1 (59.30;10.25)	7.0;3.9 (52.97;9.80)	7.2;4.7 (55.31;4.70)	7.5;2.9 (58.75;7.89)	8.8;2.2 (72.50;0.71)
Diabetes duration ≥ 5 years	80.6 (29)	75.6 (31)	84.9 (39)	83.3 (60)	83.3 (15)	81.8 (18)	100.0 (3)
Using technology for monitoring/treating diabetes	86.1 (31)	80.5 (33)	87.0 (40)	80.6 (58)	88.9 (16)	72.7 (16)	100.0 (3)
Mean (SD) number of other conditions (cancer, mental illness, late complications etc.)	1.19 (0.58)	1.54 (0.93)	1.41 (0.75)	1.57 (0.95)	1.67 (0.91)	2.36 (1.22)	1.00 (1.73)

**Other information**							

Received invitation in-person and/or needed help to answer the questionnaire	13.9 (5)	22.0 (9)	32.6 (15)	30.1 (22)	-	31.8 (7)	100.0 (3)

A line indicates < 3 observations.

SD, Standard deviation; HLQ, Health Literacy Questionnaire; eHLQ, eHealth Literacy Questionnaire; HbA1c, glycated hemoglobin.

Color coding of cells containing scale scores is based on a range of colors from green (highest) and red (lowest). Color coding does not indicate predetermined high/low scores to each scale, as they are formatted to other levels in the same row.

### Interviews

In total, 13 were conducted between February 2025 and March 2025, with characteristics presented in **Table 3**.

**Table 3:** Characteristics of Study Participants in the Interview Investigation (*N* = 13)

**Cluster**	**Age (y)**	**Sex**	**Diabetes Duration (y)**
A	60–70	Male	>5
A	60–70	Male	>5
B	50–60	Male	>5
B	50–60	Female	<5
C	60–70	Female	>5
C	30–40	Female	>5
D	50–60	Male	>5
E	50–60	Female	>5
E	60–70	Male	>5
E	60–70	Male	>5
F	80–90	Male	>5
F	30–40	Female	<5
G	70–80	Male	>5

### Vignettes

Seven vignettes were developed based on the combined findings from the cluster solutions and ID analysis.

***Cluster A: The proactive digital health strategist (n = 36).*** This cluster had the highest overall HLQ and eHLQ scores. They trust health care professionals and rely on reputable websites to guide their decisions (HLQ 1 and 8). They easily access advice and help from their social network (HLQ 4). They are digitally skilled, typically using a glucose sensor to monitor insulin levels, and they apply digital health tools effectively to manage their condition (eHLQ 1, 5 and 7).

***Cluster B: The analytical controller (n = 41).*** This cluster was characterized by individuals mainly following a diabetes-friendly diet (78%). The cluster strives for tight blood sugar control to avoid complications, which can be stressful (HLQ 3). They consult scientific journals and health care professionals to confirm their management strategies and seek advice from family (HLQ 1, 4 and 8). They use an insulin pump and have purchased a glucose sensor but have struggled to become familiar with these technologies, which can be frustrating (eHLQ 1 and 7).

***Cluster C: The practician—Support needs to be offered (n = 46).*** This cluster comprised nearly one fifth of the study population (19.3%) with more than half (54%) having a higher education. They have social support and trust health care professionals (HLQ 1 and 4). They sometimes struggle with surplus energy, which can impair their ability to maintain health routines and recall medical advice (HLQ 3). They engage with peers on social media for anonymous support, as they occasionally find it difficult to understand instructions from health care professionals (HLQ 5). Frequent sensor alarms can also cause stress in this cluster (eHLQ 7).

***Cluster D: Difficulty navigating the health system—Does not discuss illness (n = 72).*** This was the largest cluster (30%), comprising nearly one third living alone (31.9%). This cluster prefers not to talk about their illness with others and tends to manage their diabetes independently (HLQ 2 and 4). While they search for health information online, they often encounter conflicting advice and need support in interpreting it (HLQ 8 and 9). They use technology for self-management but sometimes struggle to understand the blood glucose curves in the app (eHLQ 1 and 5).

***Cluster E: Seeking a trusting health care relationship (n = 18).*** This cluster had a large proportion with lower education (55.6%). They only rely on relatives for health information but are skeptical of online sources (HLQ 2 and 8). They rarely ask health care professionals questions and struggle to discuss personal issues (HLQ 1 and 6). They use a glucose sensor and appreciate its support, though they occasionally need assistance with the app (eHLQ 7).

***Cluster F: Needs structure and support (n = 22).*** This cluster comprised 45.5% half experiencing loneliness and more comorbidity than the other cluster (2.4). They focus on blood sugar values during routine visits and have struggled with fear of hypoglycemia, feeling that their concerns were not always addressed (HLQ 1 and 3). They prefer advice from peers on social media over information from clinical websites (HLQ 8) and require support to manage appointments and medications via app (eHLQ 1 and 7).

***Cluster G: Passive with supportive spouse (n = 3, smallest cluster).*** This cluster suggests a profile consisting mainly of older individuals (mean age 73.7 years) with the highest HbA1c level across all clusters (8.8% or 73 mmol/mol), who appear to receive invitation in-person and need help to answer the questionnaire. They only visit the doctor when prompt-ed by others (HLQ 3), and their relatives might manage medical appointments, and help them recall treatment instructions (HLQ 4). They appear to avoid digital health tools, preferring paper mail out of concern for missing essential information (eHLQ 1 and 5).

## Discussion

In this study, seven distinct HL profiles were identified with 18% of participants (cluster E, F, and G) exhibiting predominantly low HL. Participants experienced greatest difficulties in navigating the health care system, indicating that individuals with T1DM generally face challenges in advocating for themselves, obtaining support to use the health care system to meet their needs, and understanding which services are available and what they are entitled to. In the context of T1DM, this pattern is particularly challenging given the high demands of technology-supported self-management and frequent interactions with outpatient care (ElSayed et al., 2025).

### Comparison With Other Studies

This study provides one of the first in-depth investigations of HL among adults with T1DM in a practice-oriented manner using vignettes. Our findings confirm the wide variation in HL profiles among individuals with T1DM, consistent with previous studies, and underscore the need for strength-based and person-centered interventions to address health inequities (Aaby et al., 2020; Esen & Aktürk Esen, 2020; Milani et al., 2025). Moreover, our results support previous findings of low HL related to navigating the health care system and appraising health information (Kasper et al., 2017). This suggests that tailoring should not only focus on individual education, but also on redesigning how services are offered, for example by simplifying access pathways for those with navigation difficulties.

## Strength and Limitations

Major strengths of this study include the design, which provides a nuanced understanding of HL, capturing both population-level variation and individual lived experience, as also demonstrated in international studies (Kelly et al., 2025). The development of text vignettes facilitated the translation of numerical data into accessible, real-world narratives, supporting interpretation (Meadows, 2022). A limitation was that the response rate of 19.9% warrants attention, as it may introduce participation bias. Compared with the Danish background population of individuals with T1DM, a greater proportion of participants in this study were above 65 years of age (26% vs. 33.6%), though the sex distribution was comparable (57% vs. 52.1%) (Danish Diabetes Database, 2024). Moreover, a higher proportion of participants had HbA1c levels ≥7% (53 mmol/mol) (33% vs. 42.4%) suggesting that the study population may represent a subgroup facing more difficulties in diabetes management, due to higher HbA1c levels than national averages (Kasper et al., 2017). This argues against the response rate necessarily reflecting a bias towards participants with higher HL. Furthermore, pooling T1DM with subgroups may have introduced heterogeneity, but the groups' shared care requirements in this setting justify the approach. While face-to-face recruitment strengthened representativeness by targeting hard-to-reach groups, the subsequent digital questionnaire may have introduced selection bias towards participants with better digital health literacy and Danish proficiency.

### Implications for Practice

Our findings underscore the need for tailored strategies in diabetes care, ensuring that support is aligned with HL. Text vignettes are practical tools to foster understanding, support codesign with patients and professionals, and stimulate reflexive dialogue about barriers and enablers of self-management. Our approach lays the foundation for the continued phases of the Ophelia process. HL assessment, combined with targeted, flexible interventions, should be prioritized to promote equitable outcomes, especially as the prevalence of T1DM continues to rise globally (Aastrup et al., 2025; Osborne et al., 2022). The findings are currently informing the subsequent codesign and implementation of contextually relevant health initiatives to enhance HL responsiveness in line with the remaining phases of the Ophelia process. Similar settings may also use these results to evaluate HL responsiveness with the goal of providing tailored and person-centered diabetes care.

## Conclusion

In this study, we identified seven distinct HL profiles among adults with T1DM, revealing significant variation in strength, needs, and preferences. A large proportion had generally low HL scores, and especially challenges in navigating the health care system were common. These findings highlight the need for HL responsive care that proactively addresses navigation challenges. By segmenting patients into distinct vignettes through cluster analysis, clinics can prioritize tailored support to improve self-management and reduce inequities among those with more challenges in HL.
